# Clinical Evaluation of Time Efficiency and Fit Accuracy of Lithium Disilicate Single Crowns between Conventional and Digital Impression

**DOI:** 10.3390/ma13235467

**Published:** 2020-11-30

**Authors:** Ji-Su Park, Young-Jun Lim, Bongju Kim, Myung-Joo Kim, Ho-Beom Kwon

**Affiliations:** 1 Department of Prosthodontics and Dental Research Institute, School of Dentistry, Seoul National University, Seoul 03080, Korea; pgs2880@snu.ac.kr (J.-S.P.); silk1@snu.ac.kr (M.-J.K.); proskwon@snu.ac.kr (H.-B.K.); 2 Dental Life Science Research Institute & Clinical Translational Research Center for Dental Science, Seoul National University Dental Hospital, Seoul 03080, Korea

**Keywords:** lithium disilicate, digital impression, time efficiency, replica technique, best fit alignment

## Abstract

The purpose of this study was to demonstrate the time-efficiency and the clinical effectiveness of chairside-fabricated lithium disilicate single crowns by digital impressions compared to the conventional method. Thirteen patients requiring a single crown on the maxillary or mandibular premolar or first molar were assigned as study subjects. The impressions were obtained using the conventional method and two digital methods with intraoral scanners: AEGIS.PO (Digital Dentistry Solution, Seoul, Korea) and CEREC Omnicam (Sirona, Bensheim, Germany). Two types of lithium disilicate single crowns were obtained; a reference crown (by conventional workflow) and a chairside crown (by digital workflow). The total time taken for fabricating the chairside crown was recorded. The replica technique was performed to compare the marginal and internal fit of the two types of crowns. In addition, accuracy of the intraoral scanners was evaluated by the best-fit alignment method. The difference between the groups was analyzed using the two-tailed paired *t*-test or one-way ANOVA, followed by the Student–Newman–Keuls test for multiple comparisons. Statistical significance was accepted at *p* < 0.05 for all statistical tests. The time required to obtain the impressions by the AEGIS (7:16 ± 1:50 min:s) and CEREC (7:29 ± 2:03 min:s) intraoral scans was significantly lower than the conventional method (12:41 ± 1:16 min:s; *p <* 0.001). There was no significant difference between the intraoral scanners. The total working time to fabricate the chairside crown averaged 30:58 ± 4:40 min:s. The average marginal gap was not significantly different between the reference (107.86 ± 42.45 µm) and chairside (115.52 ± 38.22 µm) crowns (*p >* 0.05), based on results of replica measurement. The average internal gaps were not significantly different. The average value of the root mean square between the AEGIS (31.7 ± 12.3 µm) and CEREC (32.4 ± 9.7 µm) scans was not significantly different (*p >* 0.05). Intraoral scans required a significantly shorter impression time than the conventional method, and it was possible to fabricate a lithium disilicate crown in a single visit. There were no statistically significant differences in the fit of the restorations and accuracy of the intraoral scanners compared to the conventional workflow.

## 1. Introduction 

Chairside CAD/CAM (Computer Aided Design/Computer Aided Manufacturing)procedures are more advantageous than the conventional methods in terms of fabrication efficiency of the prostheses [1,2]. Digital workflow does not require time-consuming laboratory procedures and transportation, and improves patient comfort [3,4]. The restorative materials used in the chairside CAD/CAM systems need to be milled immediately, usually within 30 min, to be delivered on the same day of tooth preparation. Manufacturers have adopted a wet grinding process on preformed blocks to minimize damage to the materials during milling and to achieve efficient post-milling time. With the exception of zirconia, which takes 6–8 h for the post-milling process, feldspathic, leucite-reinforced, lithium disilicate ceramics, and composite resin are considered for chairside CAD/CAM restorations [5].

In 2006, Ivoclar introduced IPS e.max CAD as a lithium disilicate CAD/CAM material, which has 2–3 times more flexural strength compared to the feldspathic ceramics [6]. The precrystallized block has a blue violet color and can be milled easily as a partially crystallized state. After milling, the restoration undergoes firing processes for 20–25 min in a porcelain oven under vacuum and converts to a crystallized state of lithium disilicate [7]. Accordingly, several studies have been conducted on the clinical effectiveness and long-term performance of monolithic lithium disilicate restorations. Van den Breemer et al. [8] showed that the cumulative survival rate of lithium disilicate single crowns in premolars and molars was 92% for 5 years, 85.5% for 10 years, and 81.9% for 15 years, indicating that the monolithic lithium disilicate crown is a reliable long-term clinical material. Studies that tested chairside CAD/CAM single crowns on the posterior teeth using IPS e.max CAD blocks also concluded that lithium disilicate single crowns demonstrated clinically satisfactory results [9,10].

An important factor for the longevity of dental restorations is marginal fit, which is affected by both vertical and horizontal discrepancies [11,12]. Cements can fill the marginal discrepancy; however, considering their rough and porous nature, cements can dissolve when exposed to the oral environment, resulting in microleakage and plaque accumulation [13]. Therefore, poor marginal fit of the restoration results in gingival inflammation, dental caries, and pulpal lesions [14,15]. The marginal gap is the perpendicular distance from the finish line of the prepared tooth to the internal surface of the restoration, as defined by Holmes et al. [16]. Although there is ongoing controversy regarding the acceptable values of marginal discrepancy, various studies have suggested that a 50–120 µm gap is clinically acceptable [17,18,19]. McLean and von Fraunhofer [20] conducted an in vivo study and demonstrated that restorations with cement thickness below 120 µm were more likely to succeed.

Another important factor affecting the fit of the prostheses, when deciding to use lithium disilicate as the chairside CAD/CAM material, is the intraoral scanner. Accurate digital data from intraoral scans should be the basis for favorable prostheses. Dentists can use the intraoral scanner to capture tooth surfaces and soft tissues in three dimensions to instantly analyze digital models. With the development of the chairside CAD/CAM technology, intraoral scanners have been widely used and advanced with reliable accuracy [21,22]. Direct digital impressions overcome the disadvantages of the commonly used elastomeric impression materials, including technique sensitivity, patient discomfort, dimensional changes, and laboratory errors [23,24]. However, the digital impression may also be associated with potential distortions caused by limitations in the scanning technology and accumulation of the datasets while scanning a longer arch [25,26]. To overcome these shortcomings, devices based on various non-contact optical technologies such as confocal microscopy, active stereovision, and triangulation are under development or have already been introduced in the dental market [27,28]. New digital impression techniques and devices have been compared with current reliable devices and standard processes to evaluate their accuracy and feasibility.

There are two ways to evaluate the precision of different workflows: one by comparing the fit of the resulting restorations and the other by analyzing the correspondence between the scanned and reference datasets [28]. Currently, the three-dimensional best-fit analysis is the most common and reliable method used for precision analysis of intraoral scanners. Each dataset is converted to the standard tessellation language (STL) dataset to be aligned, and the distance difference in the x, y, and z axes between the reference and test models is calculated using best-fit algorithms. The differences are represented by a color map such that the error of each area can be estimated at a glance [25,28,29]. However, research on the accuracy of the chairside CAD/CAM system remains inadequate.

The aims of this clinical study were to demonstrate whether lithium disilicate crowns fabricated with digital workflow was time-effective, compare the marginal and internal fit of lithium disilicate crowns based on direct or indirect digitalization, and to test the accuracy of the impression by comparing the laboratory scanner with two intraoral scanners.

## 2. Materials and Methods

### 2.1. Clinical Study Design

This clinical study was performed at the Department of Prosthodontics, School of Dentistry, Seoul National University, Seoul, Korea. The study protocol was approved by the Institutional Research Board (IRB No. CDE17003), and all procedures were performed according to the Declaration of Helsinki on experimentation involving human subjects (Association 2013).

The participants in need of a single crown were recruited based on the following inclusion and exclusion criteria:

Inclusion criteria:

(1) Patients aged 19–70 years;
(2) Patients in need of a tooth-supported crown in the posterior region (premolar or first molar);
(3) Presence of healthy abutment and adjacent teeth without the need for additional treatment;
(4) Normal occlusal plane of the opposite teeth;
(5) Tooth with a finishing line that could be formed supragingivally;
(6) Absence of temporomandibular or occlusal disorders;
(7) Patients who participated voluntarily in this clinical trial and signed the informed consent.

Exclusion criteria:

(1) Pregnancy;
(2) Mental illness;
(3) Allergy to the restorative material;
(4) Symptomatic teeth requiring additional endodontic treatment;
(5) Periodontally involved teeth;
(6) Presence of parafunctional habits;
(7) Inadequate crown height.

The required sample size was calculated based on the superiority test using paired *t*-test formulas [30].

$$N_{t}\;=\;\frac{\sigma_{d}{}^{2}{\left(Z_{\alpha /2}+Z_{\beta }\right)}^{2}}{d^{2}}\;≒\;9.621\;\approx \;10\;\mathrm{subject}$$

$$N\;=\;N_{t}/\left(1-0.2\right)\;=\;12.5\;\approx \;13\;\mathrm{subject}$$

where N_t_ was the number of patients in the test group, Z_α_ was the type I error (5%), Z_β_ was the type II error (20%), σ_d_ was the standard deviation of difference, and d was the mean of the difference. Zhang et al. [31] compared the superiority of the intraoral scan and plaster model with the paired *t*-test. The LR6-LL6 measurement showed a statistically significant difference in the transverse measurement in the arch; therefore, σ_d_ was 0.31 mm and d was −0.28 mm. Subject and clinical evaluation dropout rates of 10% each were estimated. Considering the total dropout rate (20%), the actual number of patients required in the test group (*N*) was 13.

A total of 15 potential participants were recruited via subway car advertising and two of the screened candidates were excluded based on the aforementioned criteria. One clinician (J.-S.P.) performed the clinical procedures. All caries and defective restorations were removed and replaced from the teeth of the participants.

### 2.2. Clinical Procedures

**Figure 1:**
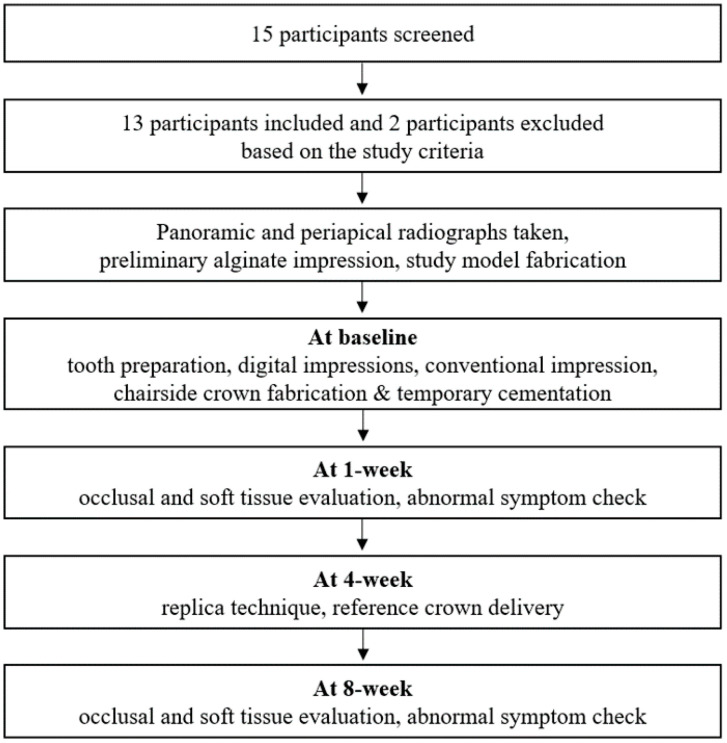
Flow-chart depicting visits, timeline, evaluation items.

Figure 1 illustrates the flow diagram of the clinical processes. Panoramic and periapical radiographs of all participants were taken. A preliminary alginate impression was made, and the study model was fabricated prior to tooth preparation.

#### 2.2.1. Tooth Preparation

The study abutment teeth were prepared to receive full-coverage ceramic crowns. The finish line was located 0.5–1.0 mm supragingivally and the preparation consisted of a shoulder margin with a rounded internal line angle. Occlusal and circumferential reductions of 1.5–2.0 mm and 1.0–1.2 mm were performed. All sharp edges were rounded off.

One conventional and two digital impressions were acquired from the prepared teeth in each of the 13 participants. The order of acquisition was as follows, first scan with AEGIS.PO (Digital Dentistry Solution, Seoul, Korea), followed by the conventional method, and finally with CEREC Omnicam (Sirona, Bensheim, Germany).

#### 2.2.2. Conventional Impression

Since the abutment finish line was formed 0.5–1.0 mm above the gingival line, all impressions were obtained without cord insertions. Impression of the associated quadrant was obtained using a perforated plastic ready-made tray. VPS tray adhesive (Kerr, Romulus, MI, USA) was applied to the tray. The impression was made using light-body polyvinyl siloxane (Imprint II Garant; 3M ESPE, Seefeld, Germany) and putty (Exaflex Putty; GC, Tokyo, Japan) using a one-step technique. The impression material was set in the patient’s mouth considering the safety time recommended by the manufacturer. Impression of the opposing jaw was obtained using alginate (Aroma fine plus; GC, Tokyo, Japan) and the interocclusal record was acquired using polyvinyl siloxane (O-bite; DMG, Hamburg, Germany). The time taken to obtain the impression was recorded using a digital stopwatch (HS3V-1B; Casio Computer Corp., Seoul, Korea). The VITA classic shade guide (VITA Zahnfabrik, Bad Säckingen, Germany) was used to obtain the shade for the crowns.

#### 2.2.3. Digital Impressions

Two digital systems, namely the AEGIS.PO and CEREC Omnicam, were tested to obtain intraoral optical impressions. The intraoral scanner was calibrated before each patient was scanned. Quadrant scans were performed, and the scan sequences were chosen according to the manufacturer’s guidelines. The abutment, antagonist, and interocclusal records were scanned with the AEGIS and CEREC systems. Before scanning with AEGIS, a VITA powder scan spray (VITA Zahnfabrik, Bad Säckingen, Germany) was applied to the tooth surface. Each scan time was measured with a digital stopwatch.

**Figure 2:**
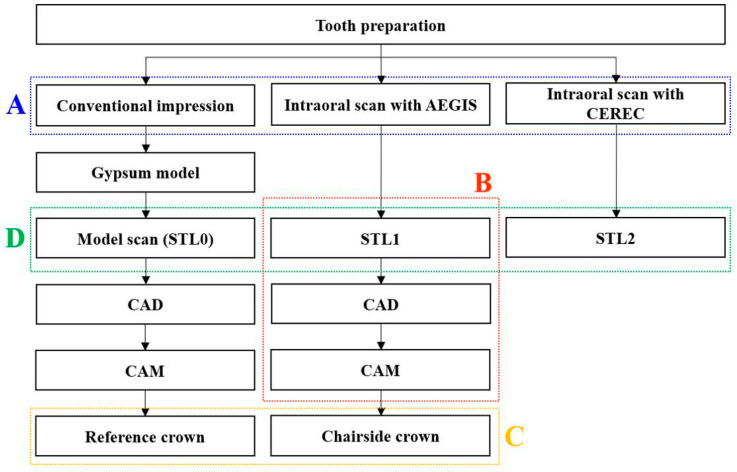
Overview of this study, **A**, Impression time; comparison of the time taken to obtain impressions by the conventional and intraoral scanning methods, **B**, Total working time; total working time for fabricating the chairside crown, **C**, Replica measurement; evaluation of marginal and internal fit of the two crowns, **D**, Best fit alignment; evaluation of the accuracy of intraoral scanners.

**Figure 3:**
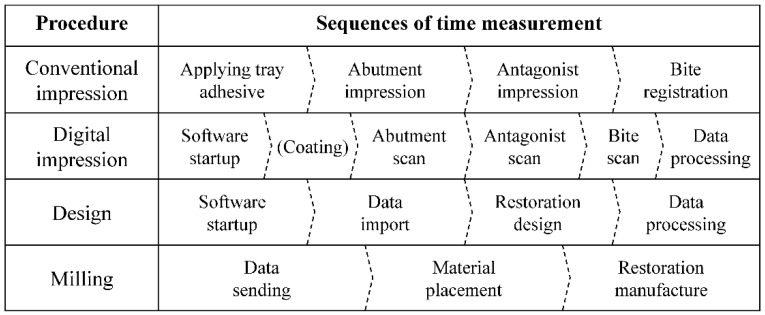
Specific steps in the procedure for time measurement.

The time taken to obtain one conventional impression and two digital impressions were assessed (Figure 2A). The time needed to obtain the conventional impression was recorded from the beginning of application of the tray adhesive to the end of the bite material removal from the patient’s mouth. The time taken to obtain digital impressions was recorded from software startup to data processing (Figure 3).

#### 2.2.4. Chairside Crown Fabrication

**Figure 4:**
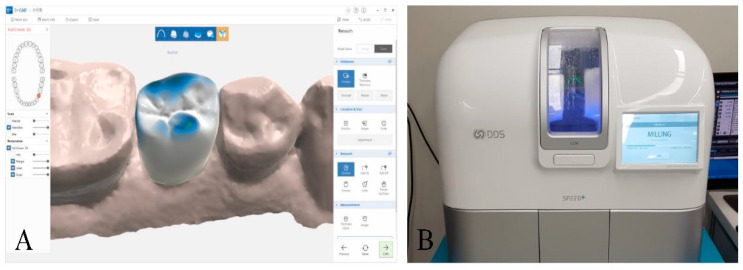
Chairside CAD/CAM procedures. (**A**) Planning a chairside crown with the design software. (**B**) Manufacturing a lithium disilicate crown with the milling machine.

Using the file from the AEGIS intraoral scanner, a lithium disilicate crown was designed using the design software (DESIGN+ Suite, Digital Dentistry Solution, Seoul, Korea; Figure 4A). All crowns were designed with the same settings (cement gap: 70 µm, layer thickness: 600 µm, edge reinforcement: 200 µm). The design file was sent to the chairside milling machine (SPEED+, Digital Dentistry Solution, Seoul, Korea), and single crowns were fabricated with lithium disilicate glass-ceramic blocks (IPS e.max CAD; Ivoclar Vivadent, Amherst, NY, USA; Figure 4B). Each design and milling time was recorded with a digital stopwatch and the total time for digital workflow was investigated (Figure 2B and Figure 3).

After the final sintering procedure, the crowns were tried intraorally and adjusted if necessary, except for the internal surface, and cemented with a eugenol-free temporary cement (Tempbond NE; Kerr, Romulus, MI, USA). All procedures were performed in one treatment appointment.

#### 2.2.5. Reference Crown Fabrication and Delivery

Conventional impression materials were disinfected and type IV dental stone (Fujirock; GC, Tokyo, Japan) was poured. The master casts were scanned with a laboratory scanner (Identica Hybrid; Medit, Seoul, Korea). Subsequently, the restorations were designed using the DESIGN+ Suite software with the same settings as the chairside CAD process. IPS e.max CAD blocks were milled using SPEED+ machine and all laboratory procedures were performed by a single technician.

Four weeks after preparing the abutment, a replica technique [20,32,33] was performed to register the marginal and internal fit of the two groups (Figure 2C). A reference crown (control group) was obtained by conventional workflow and a chairside crown (test group) was obtained by digital workflow. The internal surface of the crowns was filled with low-viscosity silicone (Fit-checker II; GC, Tokyo, Japan) and seated on the abutment tooth by applying maximum finger pressure for 3 s. The patients were continuously instructed to bite on the cotton roll. The crowns were carefully removed after 2 min with the silicone film adherent to the internal surface. The film was stabilized by injecting a light-body polyvinyl siloxane (Examixfine Injection type; GC, Tokyo, Japan). After setting of the second material, the base of the replica was reinforced with heavy-body polyvinyl siloxane (Imprint II Garant; 3M ESPE, Seefeld, Germany).

Subsequently, a reference crown fabricated by the conventional impression method was cemented using a dual-cure resin cement (Variolink N; Ivoclar Vivadent AG, Schaan, Liechtenstein) according to the manufacturer’s guidelines.

### 2.3. Replica Measurement

**Figure 5:**
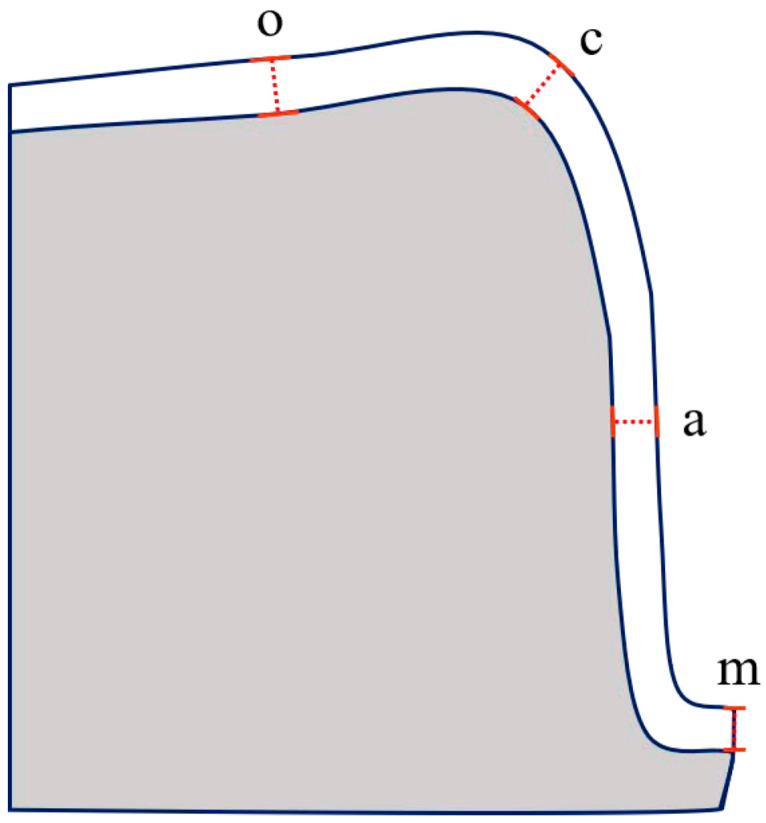
Schematic diagram of the points measured on each cross-section, o, occlusal gap (midpoint of occlusal wall); c, gap of cusp tip (axio-occlusal transition point); a, axial gap (midpoint of axial wall); m, marginal gap.

Each replica was sectioned into four parts, buccolingually and mesiodistally, using a sharp scalpel. The thickness of the replica film corresponding to the discrepancy between the crown and the abutment tooth was measured using a stereomicroscope (SMZ168, Motic, Wetzlar, Germany) at 50× magnification. The specimen images were transferred to an analysis software (Motic Images Plus 3.0, Motic, Wetzlar, Germany) using a digital microscope camera (Moticam 3+, Motic, Wetzlar, Germany). The widths of the marginal, axial, cuspal, and occlusal areas were measured on all aspects of the sectioned replica (Figure 5).

**Figure 6:**
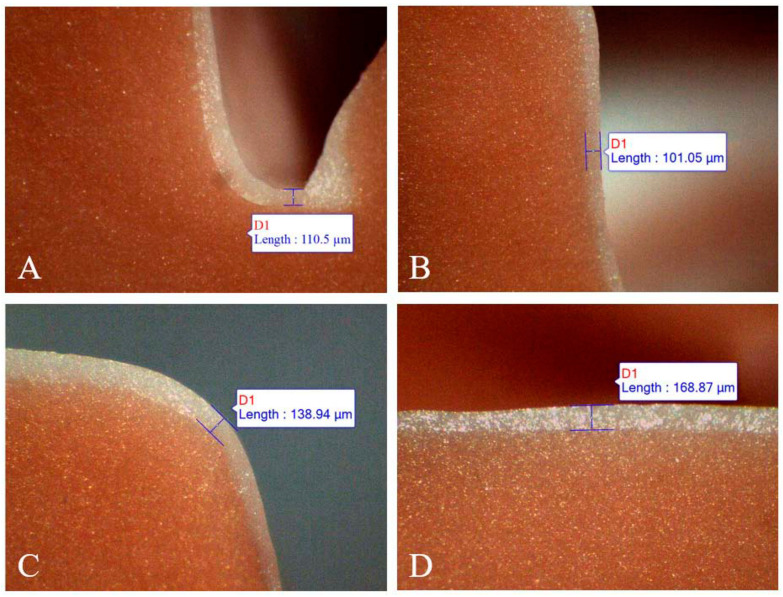
The blue lines represent the measurement discrepancies between the crown and the abutment tooth by the analysis software. **A**, marginal gap, **B**, axial gap, **C**, gap of the cusp tip, **D**, occlusal gap.

The marginal gap was recorded as the perpendicular distance from the finish line of the prepared tooth to the internal surface of the restoration, as defined by Holmes et al. [18]. The internal gaps were measured at the midpoint of each wall (Figure 6). All replicas were cut and measured by the same trained operator.

### 2.4. Best-Fit Alignment

**Figure 7:**
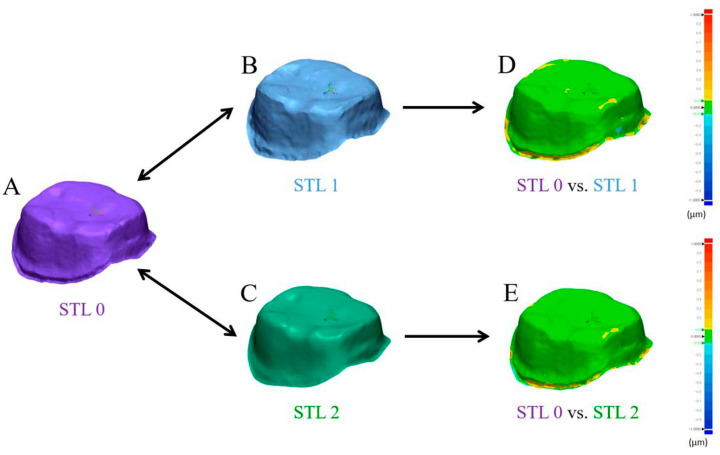
Simplified graphic representation of the analysis by best-fit alignment, **A**, Scan data of the gypsum model by laboratory scanner, **B**, Scan data using the AEGIS intraoral scanner, **C**, Scan data using the CEREC intraoral scanner, **D**, Deviation after superimposition of A and B displayed in a color-coded map. **E**, Deviation after superimposition of A and C displayed in a color-coded map.

Accuracy of the intraoral scanners was compared by overlaying the scan images obtained with AEGIS (STL1) and CEREC (STL2) with those of the laboratory scanner (STL0; Figure 2D). Each dataset was converted to an STL format and then imported into the 3D analysis software (Geomagic Control X; 3D Systems, Rock Hill, SC, USA). The datasets were trimmed to the field of interest, including the prepared area of the abutment teeth and all artifacts and unrelated areas below the preparation lines were eliminated by the 3D modeling software (Rhinoceros 6.0; Robert McNeel and Associates, Seattle, WA, USA). The trimmed datasets from STL1 and STL2 were separately superimposed with the STL0 dataset using a best-fit algorithm (Figure 7). The software calculated the three-dimensional divergences between each test and reference dataset, and produced results of mean positive and negative deviations and root mean square values. The 3D differences were represented by a color-coded image.

### 2.5. Statistical Analysis

Descriptive statistics were computed for all variables with software (Sigma Plot 14.0, Systat Software Inc., San Jose, CA, USA). As the data were normally distributed (Shapiro–Wilk test), differences between the groups were analyzed using two-tailed paired *t*-test or one-way ANOVA, which was followed by the Student–Newman–Keuls test for multiple comparisons. The data distributions were represented with boxplots, and the data were reported using means, standard deviations (SD), ranges, and 95% confidence intervals. Statistical significance was accepted at *p* < 0.05 for all statistical tests.

## 3. Results

Thirteen patients (4 men and 9 women) participated in the study. The mean age of the patients was 49.0 ± 13.4 (range, 22–67) years. Two maxillary second premolars, three maxillary first molars, four mandibular second premolars, and four mandibular first molars were treated.

### 3.1. Comparison of Time Taken for Impression

**Figure 8:**
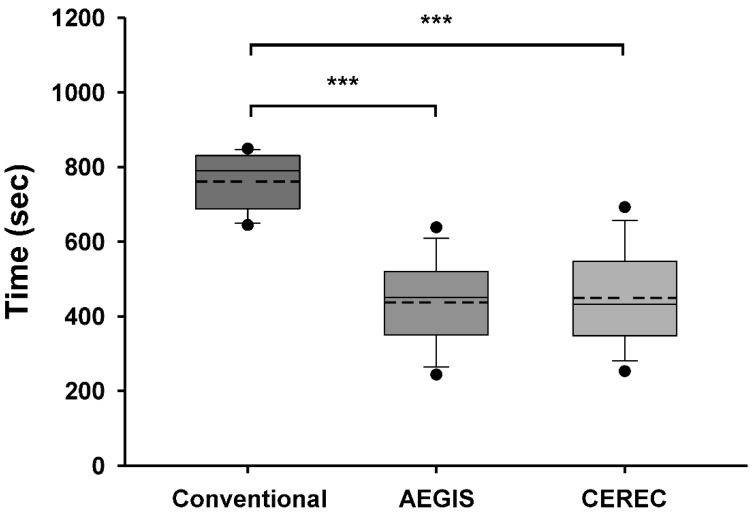
Box plot of the time required for each impression method (***, *p <* 0.001).

The mean time taken to obtain the impression was 12:41 ± 1:16 min:s by the conventional method, 7:16 ± 1:50 min:s by the AEGIS intraoral scan, and 7:29 ± 2:03 min:s by the CEREC intraoral scan. The average time taken to obtain the impressions by the AEGIS and CEREC intraoral scans were significantly lower than that taken by the conventional method (*p <* 0.001; Figure 8). The difference between the AEGIS and CEREC intraoral scans was not statistically significant (*p* = 0.767).

### 3.2. Total Working Time for the Chairside Crown by the AEGIS Stystem

The milling process took the longest time on average among the procedures for fabricating the chairside crown, and the mean scan time was longer than the mean design time. The total working time for scanning, designing, and milling averaged 30:58 ± 4:40 min:s. The maximum total working time was 39:16 min:s (Table 1).

### 3.3. Marginal and Internal Fit

**Figure 9:**
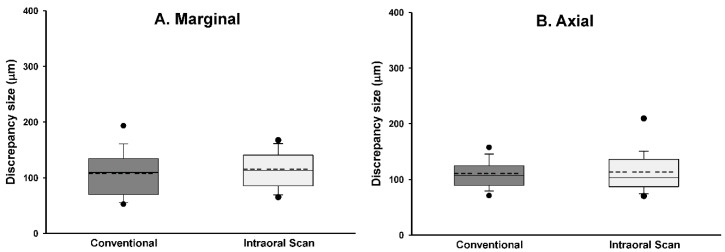

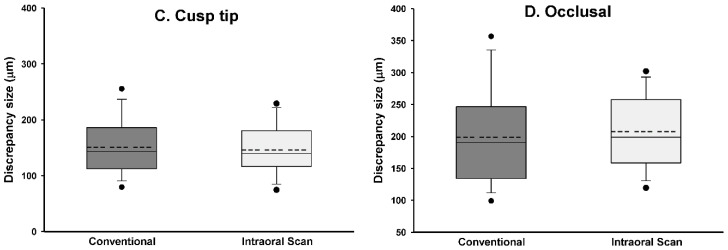
Box plots of discrepancies in the four regions of interest, (**A**) marginal discrepancy, (**B**) axial discrepancy, (**C**) discrepancy in the cusp tip, and (**D**) occlusal discrepancy.

The replica measurements, including the mean, standard deviation, median, 95% confidence interval, and maximum and minimum values for both the reference and the chairside crowns are presented in Table 2. The Shapiro–Wilk test revealed a normal distribution of the data in the two groups (*p >* 0.05). The mean marginal gap in the conventional workflow was lower than that in the chairside workflow; however, the difference was not statistically significant (*p >* 0.05). The average internal gaps, including those of the axial wall, cusp tip, and occlusal regions, were not significantly different between the two workflows (*p >* 0.05). Analyses of the significances between the regions revealed significant differences in all correlations, with the exception of the marginal gap with the axial gap *(p <* 0.001; Table 3; Figure 9A–D).

### 3.4. Accuracy of Intraoral Scanners

**Figure 10:**
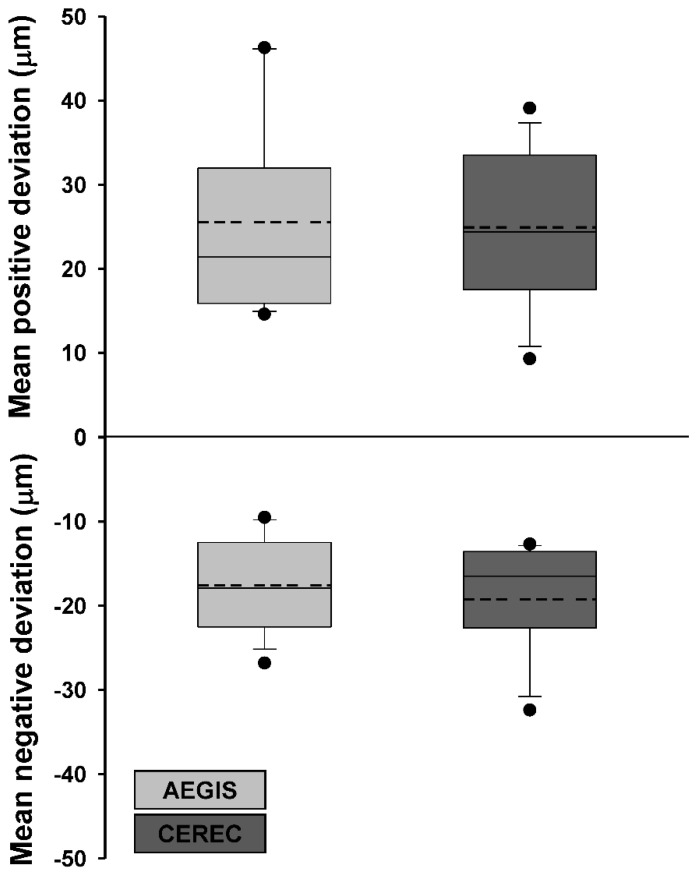
Box plot of the mean positive and negative deviations after superimposition of the CI dataset with datasets obtained from the AEGIS and CEREC scans.

**Figure 11:**
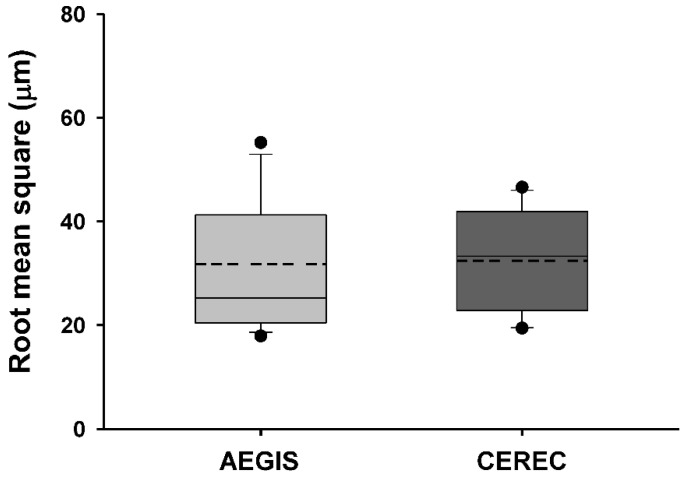
Box plot of root mean square between the CI dataset and datasets obtained using the AEGIS and CEREC scans.

**Figure 12:**
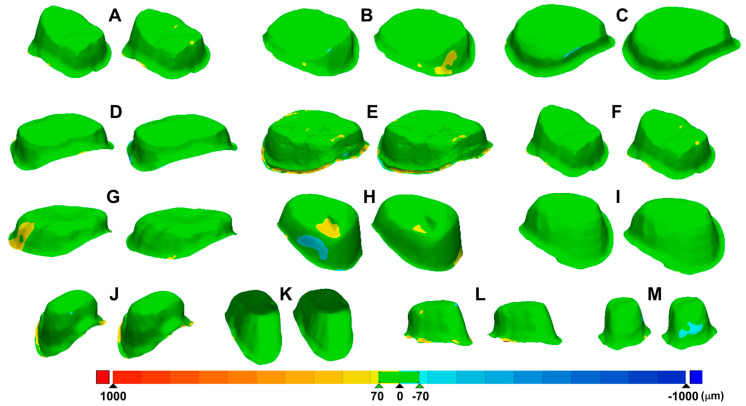
**A**–**M** (left: AEGIS, right: CEREC), all 13 datasets generated by overlapping STL0 and STL1.

Table 4 presents the average and standard deviation values of the mean positive and negative deviations and the root mean square of each experimental dataset after superimposition with the reference dataset. The Shapiro–Wilk test revealed that the two test groups had a normal distribution (*p >* 0.05). However, none of the parameters between the AEGIS and CEREC scans were significantly different (*p >* 0.05; Figure 10 and Figure 11). Figure 12 shows the color-coded maps, which represents 3D differences after each superimposition of all abutment teeth.

## 4. Discussion

Comparison of the working time according to the impression methods used in this study revealed that the conventional method took significantly longer time than the intraoral scan method. In general, the scan range for a digital impression of a single abutment is limited to a quadrant in the arch. Thus, even in the conventional method, impression of the quadrant, rather than that of the full arch, was obtained using the partial tray. Therefore, the conventional method was not disadvantageous compared to the intraoral scan method.

Nevertheless, similar to the results of previous studies, more time was taken to obtain the impressions by the conventional method than the intraoral scan method. Yuzbasioglu et al. [3] and Schepke et al. [34] compared the time taken to obtain conventional and digital impressions of the complete arch. The studies used the CEREC Omnicam intraoral scanner to obtain the digital impressions. Both in vivo studies concluded that the digital impression technique was more time-efficient than the conventional method. Gjelvold et al. [35] evaluated the time taken to obtain impressions of tooth-supported single crowns. The mean impression times by the digital and conventional techniques were 7:33 min:s and 11:33 min:s, respectively. The study demonstrated that the digital technique took significantly less time than the conventional technique. In this study, one clinician trained in scanning devices could standardize the procedure of the abutment scan and time for the measurement, whereas inexperienced users may take longer to achieve the same. Lee and Gallucci [36] evaluated the time efficiency of digital and conventional impression techniques in a single implant restoration model. Novice users took 24:43 min:s by the conventional approach and 12:29 min:s by the digital approach. Thus, the digital impression technique was significantly faster even among inexperienced users.

The conventional process requires time for the preparation of impression trays and the setting time of impression materials is standardized. Furthermore, patients prefer the intraoral scan than the conventional impression method [3,4,34,35]. Patient discomfort associated with the conventional impression technique might influence the working time. However, fewer variables are involved in the clinical process of the conventional impression technique, thus the associated standard deviation may be small.

The AEGIS scan was marginally faster than the CEREC scan; however, there was no significant difference. The difference in scan times between intraoral scanners is related to the different scan software and hardware capabilities. In addition to the size of the scanned data associated with resolution of the device, the process of modifying, converting, and compressing the images in the software affects the scan time.

The chairside CAD/CAM system of the Digital Dentistry Solution company is an all-in-one system to scan, design, and manufacture crowns, similar to CEREC chairside solutions. Studies reporting on the total working time of the chairside CAD/CAM system are limited. In the present study, the total working time to fabricate the chairside crown, combined with the scanning, designing, and milling times averaged 30:58 min:s, and the maximum time taken was 39:16 min:s. The post-milling process was the same for all crowns, which required a constant 20 min in the ceramic furnace. The total time for scanning, designing, milling, and post-milling processes is expected to be approximately 51 min and does not exceed 60 min. However, the total time for chairside crown delivery should include the times for tooth preparation, adjustment, and cementation. Nonetheless, standardization of these processes appears difficult due to involvement of several variables depending on patient management, abutment status, clinical situations, and the ability of the operator. The fact that the time required for the chairside process was within 60 min supports the possibility of delivering the crown in one day in the dental clinic.

In clinical studies, the quality of adaptation of the restoration can be estimated by intraoral radiographs, tactile evaluation, and the replica technique [20,37,38]. In particular, the replica technique involves measuring a silicone replica of the space between the tooth and the restoration using a microscope, and allows reliable prediction of the cement thickness regardless of the location [32,39,40].

The majority of the literature published before 2014 argued that the marginal fit of conventionally fabricated lithium disilicate crowns was better than crowns fabricated with the CAD/CAM technology [41]. However, papers published after 2014 showed a tendency of improved marginal fit of CAD/CAM crowns. Several studies that compared lithium disilicate crowns showed no significant differences in the marginal discrepancy and internal discrepancy volume between the conventional and CAD/CAM methods [42,43,44,45]. An in vitro study by Alfaro et al. [46] that measured the internal adaptation of lithium disilicate crowns by a micro-CT scan, reported that CAD/CAM crowns fabricated by the Lava COS digital impression system demonstrated better internal fit than those fabricated by the conventional impression technique. Haddadi et al. [47] conducted an in vivo study and reported that the marginal and internal gaps in CAD/CAM crowns were significantly lower than those in conventional crowns in all regions, except the cusp tip.

In recent in vitro trials, lithium disilicate crowns fabricated by the conventional and CAD/CAM methods showed no significant differences in the marginal discrepancy [42,43,44]. In a clinical study, Berrendero et al. [48] compared the marginal discrepancy of all-ceramic crowns fabricated by the conventional and digital impression techniques. The study used the Trios scanner for intraoral scans, and no statistically significant differences were found between the conventional method (119.9 ± 59.9 µm) and the digital method (106.6 ± 69.6 µm). Zeltner et al. [45] evaluated the marginal fit of lithium disilicate single crowns based on a conventional workflow and four digital workflows. They found that the average marginal discrepancies were 90.4 ± 66.1 µm by the conventional workflow, 83.6 ± 51.1 µm by the CEREC infinident workflow, 94.3 ± 58.3 µm by the Lava COS scanner, 127.8 ± 58.3 µm by the iTero scanner, and 141.5 ± 106.2 µm by the CEREC inLab workflow. There were no significant differences between the conventional and digital workflows. Other studies have argued that crowns fabricated using the intraoral scanning technique had better marginal fit than those by the conventional impression technique [47,49].

Previous investigations have demonstrated that marginal discrepancies below 120 µm are clinically acceptable [17,18,19,20]. Our results showed that both crowns fabricated by the conventional and digital workflows had a good marginal adaptation. The reference crowns were cemented after 4 weeks considering the time taken for transportation and laboratory procedures. Moreover, the delayed cementation was to eliminate potential symptoms of the abutment and adjacent teeth and to allow sufficient adaptation of the soft tissues. At 8 weeks, all crowns were clinically evaluated and no specific symptoms were observed.

Although the mean marginal gap of the reference crown was slightly smaller than that of the chairside crown, there was no statistically significant difference. In the present study, one dental technician used the same CAD/CAM system with standardized design and milling parameters for all restorations. The number of rescans and errors can be reduced in the case of a single crown restoration. Hence, the digital workflow enables a favorable crown fit similar to the conventional workflow.

There were no significant differences in the internal discrepancies between the two groups. Both the axial and occlusal gaps in the reference crown were smaller than that in the chairside crown, as was the marginal gap. However, the cuspal gap in the reference crown was larger than that in the chairside crown. The marginal differences could have originated from the performance of the laboratory or the intraoral scanners because the same CAD/CAM system was used to fabricate the two types of crowns. Several errors could occur in the cusp tip area during scanning, which has more sharp angles compared to other parts. Furthermore, accurate reproduction of the internal surface was difficult using the milling bur. The occlusal mean value of the internal gaps was the largest in the crowns fabricated by both methods due to accumulation of errors such as the undercut of the axial wall and incomplete crown seating. Meanwhile, the axial wall is often smooth and straight without an angle. These characteristics facilitate scanning of the axial gap and result in the smallest value among the internal gaps.

Intraoral digitization is a step to prevent possible errors in advance at the beginning of the digital workflow. Our clinical study sought to evaluate datasets from two intraoral scanners by superimpositions. We adopted the “best fit alignment” methodology to overlap the test groups with the reference dataset. Best-fit alignment has already been used in other studies to compare 3D datasets [50,51,52]. Güth et al. [28] compared the accuracy of direct and indirect digitalization approaches using the best-fit methodology. This in vitro study reported that the mean absolute values of Euclidean distances were 15 ± 6 µm for direct digitalization and 36 ± 7 µm for indirect digitalization. Nedelcu et al. [53] performed best-fit alignment to compare the accuracy and precision of three intraoral scanners and the conventional impression method. In best-fit analysis, the mean positive and negative deviations and the root mean square value describe the spatial proximity between the test object and reference.

The term “accuracy” in this study actually meant trueness. Accuracy generally represents both trueness and precision [54]. Identica Hybrid scanner was used to obtain a true value to evaluate the two intraoral scanners. The laboratory scanner has a high accuracy of less than 7 µm and is popularly used in dental offices for various restorations [55,56]. There was no statistically significant difference in the AEGIS (STL1) and CEREC (STL2) datasets compared to the STL0 dataset. However, the absolute value of the mean positive deviation of AEGIS was higher than that of CEREC, and the absolute value of the mean negative deviation of CEREC was higher than that of AEGIS. This result indicated that the AEGIS dataset had more protruded surfaces and the CEREC dataset had more depressed surfaces compared to the reference dataset. AEGIS was a powder-type intraoral scanner, whereas CEREC was a powder-free intraoral scanner. Although the thickness of the powder was negligible, the use of powder may have influenced the increase in the positive deviation and decrease the negative deviation of the AEGIS dataset.

The root mean square value reflecting both deviations is related to the absolute Euclidean distance. There was no significant difference between the groups. Nevertheless, the root mean square value of the AEGIS dataset was marginally lower than that of the CEREC dataset. The two intraoral scanners used in this study work on different image-capturing technologies. The AEGIS scanner realizes a model by photographing still cuts under the principle of the stereo-structured light, whereas the CEREC scanner records a color image and a video of objects using optical triangulation and confocal microscopy [27,29]. Several in vivo and in vitro studies have reported a substantial difference in the accuracy of intraoral scanners from different manufacturers [29,45,57,58]. The marginal difference in the accuracy between the two scanners might be attributed to the different working principles.

Regarding the design of the present study, abutment teeth that could have a supragingival margin for favorable accessibility were mainly chosen. Our observations must be applied with caution in more challenging clinical situations. Since this study was limited to a single crown, further studies involving fixed partial dentures or full arches that comprise multiple abutments are necessary. Although the replica technique is a proven method for measuring the fit, it is technique sensitive and has a limited number of sections. Lastly, errors may occur when comparing the accuracy of intraoral scanners due to differences in the resolution.

## 5. Conclusions

The present study demonstrated the time-efficiency and the clinical effectiveness of lithium disilicate single crowns fabricated at chairside by digital impressions compared to the conventional impression technique. Within the limitations of the present clinical study, intraoral scans demonstrated a significantly shorter impression time than the conventional method. The clinical implication is that the total working time to fabricate the chairside crown was up to 40 min. Thus, it is possible to fabricate a lithium disilicate crown in a single dental visit. The marginal discrepancy of the chairside crown was within the clinically acceptable range. There were no statistically significant differences in the fit of the restorations and accuracy of the intraoral scanners compared to the conventional workflow.

## Figures and Tables

**Table 1:** The time required for each step and the total working time for chairside fabrication of the single crowns by the AEGIS system.

	Scan (min:s)	Design (min:s)	Milling (min:s)	Total (min:s)
**Mean**	07:16	06:50	16:51	30:58
**Maximum**	10:38	12:20	22:00	39:16
**Minimum**	04:03	04:28	11:38	24:33
**SD**	01:50	02:15	03:30	04:40

SD, standard deviation.

**Table 2:** Results of the replica measurements in micrometers at four locations: marginal gap, axial wall, cusp tip, and occlusal gap.

Region	Method	Mean ± SD (Median)	95% Confidence Interval (Range)	Normality	*p* Value
**Marginal**	CI	107.86 ± 42.45 (109.13)	82.21–133.51 (49.87–219.90)	0.544	0.381
	IOS	115.52 ± 38.22 (112.64)	92.42–138.62 (27.08–244.32)		
**Axial**	CI	110.84 ± 33.43 (107.14)	90.64–131.05 (67.07–276.76)	0.057	0.582
	IOS	113.05 ± 35.67 (102.98)	91.50–134.61 (57.90–219.34)		
**Cusp**	CI	151.04 ± 52.58 (143.32)	119.27–182.81 (71.37–294.22)	0.452	0.577
	IOS	146.38 ± 46.78 (139.83)	118.11–174.65 (58.77–246.93)		
**Occlusal**	CI	198.92 ± 77.04 (190.66)	152.37–245.48 (96.81–386.78)	0.053	0.442
	IOS	207.54 ± 60.42 (198.84)	171.03–244.05 (101.82–344.20)		

CI: Conventional Impression, IOS: Intraoral Scan.

**Table 3:** Significances between regions of the replica.

Region	Marginal	Axial	Cusp	Occlusal
**Marginal**	CI IOS	*p* = 0.800 *p* = 0.278	*p* < 0.001 *p* < 0.001	*p* < 0.001 *p* < 0.001
**Axial**	*p* = 0.800 *p* = 0.278	CI IOS	*p* < 0.001 *p* < 0.001	*p* < 0.001 *p* < 0.001
**Cusp**	*p* < 0.001 *p* < 0.001	*p* < 0.001 *p* < 0.001	CI IOS	*p* < 0.001 *p* < 0.001
**Occlusal**	*p* < 0.001 *p* < 0.001	*p* < 0.001 *p* < 0.001	*p* < 0.001 *p* < 0.001	CI IOS

CI: Conventional Impression, IOS: Intraoral Scan.

**Table 4:** Results (in micrometers) obtained by superimposition of the CI dataset with datasets obtained using the AEGIS and CEREC scans.

Group	AEGIS	CEREC	Normality	*p* Value
	Mean ± SD	Mean ± SD		
**Mean positive deviation**	25.5 ± 11.0	24.9 ± 9.2	0.774	0.738
**Mean negative deviation**	−17.6 ± 5.4	−19.2 ± 6.2	0.262	0.409
**Root mean square**	31.7 ± 12.3	32.4 ± 9.7	0.894	0.760

SD, standard deviation.
